# Clear-Cell Sarcoma With an Unusual Presentation Mimicking Metastatic Melanoma

**DOI:** 10.7759/cureus.32010

**Published:** 2022-11-29

**Authors:** Layla Tahiri Elousrouti, Nawal Hammas, Fatima zahra Elmernissi, Hinde Elfatemi, Laila Chbani

**Affiliations:** 1 Department of Pathology, Hassan II University Hospital, Biomedical and Translational Research Laboratory, Faculty of Medicine and Pharmacy, Sidi Mohamed Ben Abdellah University, Fes, MAR; 2 Department of Dermatology and Venerology, Hassan II University Hospital, Fes, MAR

**Keywords:** case report, immunohistochemistry(ihc), fluorescence in situ hybridization, cutaneous, melanoma, clear cell sarcoma

## Abstract

Clear-cell sarcoma (CCS) was first described in 1968. It partly overlaps morphologically, immunohistochemically, and ultrastructurally with malignant melanoma (MM), hence its name "soft tissue melanoma." Nevertheless, there are sufficient cytogenetic differences between cutaneous melanoma and clear-cell sarcoma to consider clear-cell sarcoma as a separate entity. Clear-cell sarcoma of soft tissue is different from clear-cell sarcoma of the kidney. It is classified as a tumor of uncertain differentiation in the WHO 2020 classification of soft tissue tumors. It is an aggressive, rare malignant tumor that is involved in the deep soft tissues of the extremities and trunk. We report a case of primary clear-cell sarcoma of unusual presentation in a 31-year-old young man, mimicking metastatic melanoma.

A 31-year-old man presented with a heel mass of 2.5 cm. Histologically, it was a dermal and hypodermal nodular proliferation of spindle cells of 23 mm with a grenz zone under the epidermis. There was no necrosis area or lymphovascular invasion. Surgical margins were free. There were no clinically suspicious lymph nodes. The tumor cells were stained for S100 protein, MELAN A, and HMB45, which led to an initial diagnosis of metastatic malignant melanoma. However, analysis by fluorescence in situ hybridization (FISH) found a rearrangement of the Ewing sarcoma region 1 (EWSR1)* *gene, which led to a diagnosis of primary clear cell sarcoma in the skin.

This case highlights the importance of considering the diagnosis of a clear-cell sarcoma in front of any dermal lesions with morphological and immunohistochemical melanocytic features that do not have an in situ component and of atypical presentation, especially in young patients, hence the interest in performing fluorescence in situ hybridization for EWSR1, which remains the key to the diagnosis of cutaneous clear-cell sarcoma.

## Introduction

Clear-cell sarcoma (CCS) of soft tissue is a malignant tumor involving deep soft tissue and is characterized by Ewing sarcoma region 1 (EWSR1) rearrangement. It overlaps morphologically, immunohistochemically, and ultrastructurally with malignant melanoma (MM), hence its name "soft tissue melanoma," which is not recommended [1,2]. It is classified as a tumor of uncertain differentiation in the WHO 2020 classification of soft tissue tumors [1]. Clear-cell sarcoma occurs most commonly at deep sites in the extremity, in the periarticular regions, with 50% of cases arising in the lower extremities, where they can be associated with tendons or aponeuroses [2]. There are also primary clear-cell sarcomas of the digestive tract, the lung, and the mediastinum in the head/neck region [3]. Lesions arising in the skin are recognized in the literature [4]. Dermis-localized clear-cell sarcoma is rare, and few cases have been reported [2]. Due to the rarity of this neoplasm, the tumor can be confused with cutaneous melanoma since they share similar pathologic and immunohistochemical characteristics. Although clear-cell sarcoma is considered the soft tissue counterpart of melanoma, it can be genetically differentiated from cutaneous melanoma. Cases of malignant melanoma may contain BRAF mutations, whereas clear-cell sarcoma lacks this mutation [5] and characteristically exhibits the reciprocal translocation t(12;22)(q13;q12), which can be detected by using a dual-color, break-apart fluorescence in situ hybridization (FISH) probe, allowing for the detection of EWS (22q12) gene rearrangement in formalin-fixed, paraffin-embedded tissues [6]. We report a case of primary clear-cell sarcoma of unusual presentation in a 31-year-old young man, mimicking metastatic melanoma.

## Case presentation

**Figure 1:**
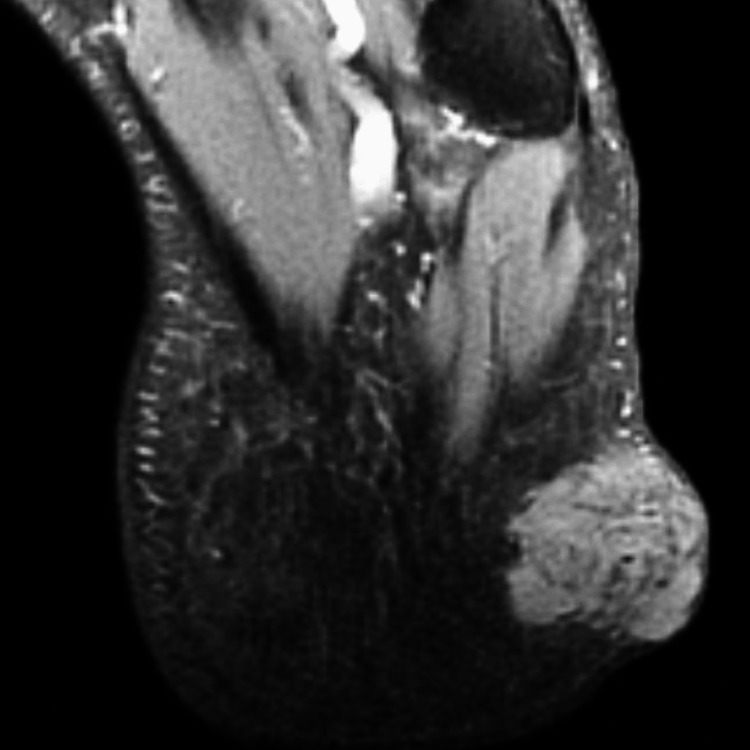
MRI T2 proton density showing subcutaneous well circumscribed tumor.

**Figure 2:**
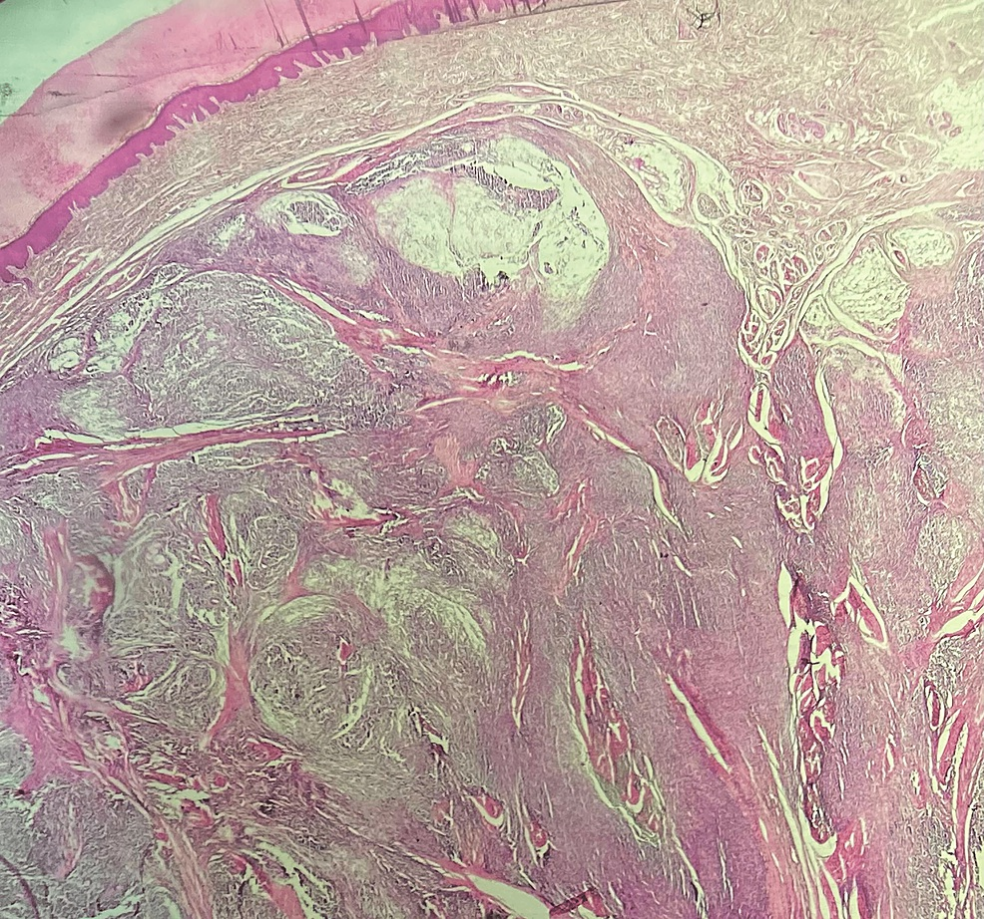
Dermal nodular mass with fascicles of spindle cells and cellular nests separated by fibrous bands. No epidermal component was found with a grenz zone (HES ×40).

**Figure 3:**
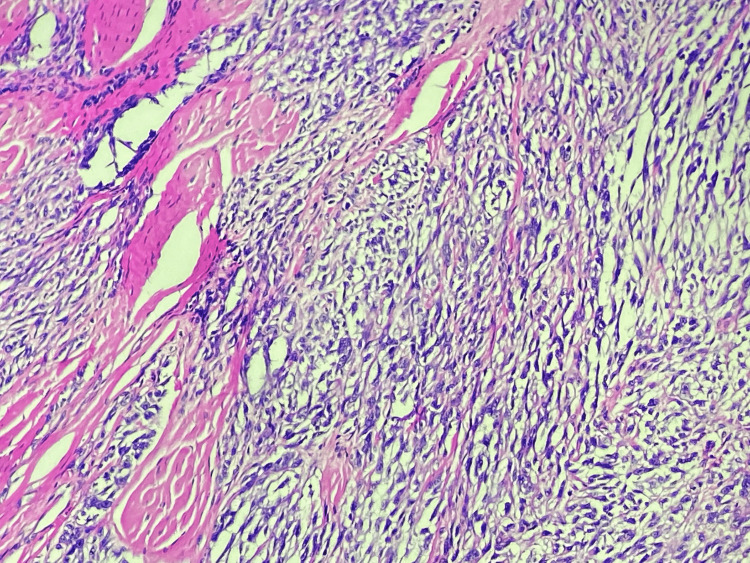
Cells are round and fusiform showing vesicular nuclei, prominent nucleoli, and clear-to-pale eosinophilic cytoplasm (HES ×200).

**Figure 4:**
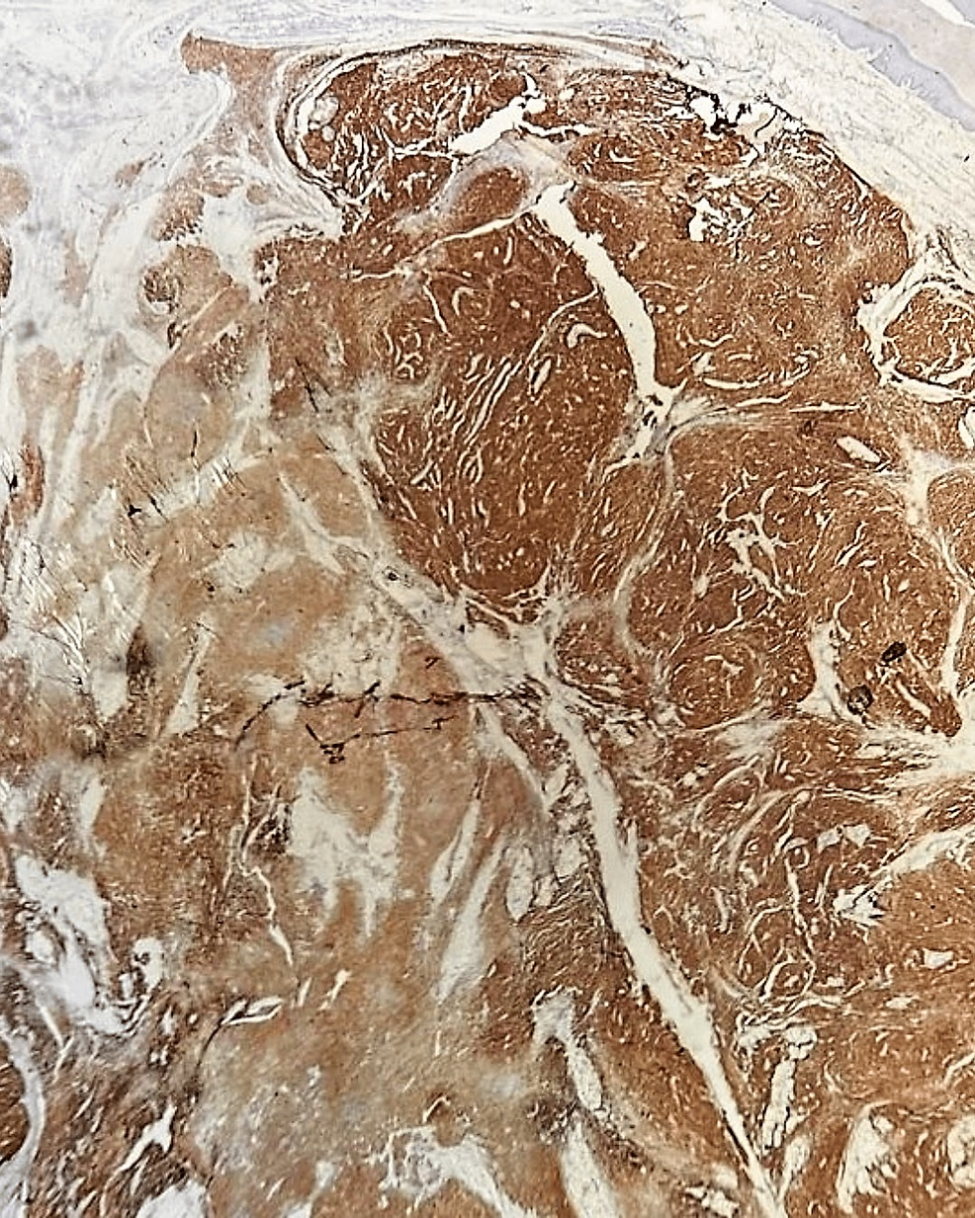
Immunostaining with HMB45 which is diffusely positive.

**Figure 5:**
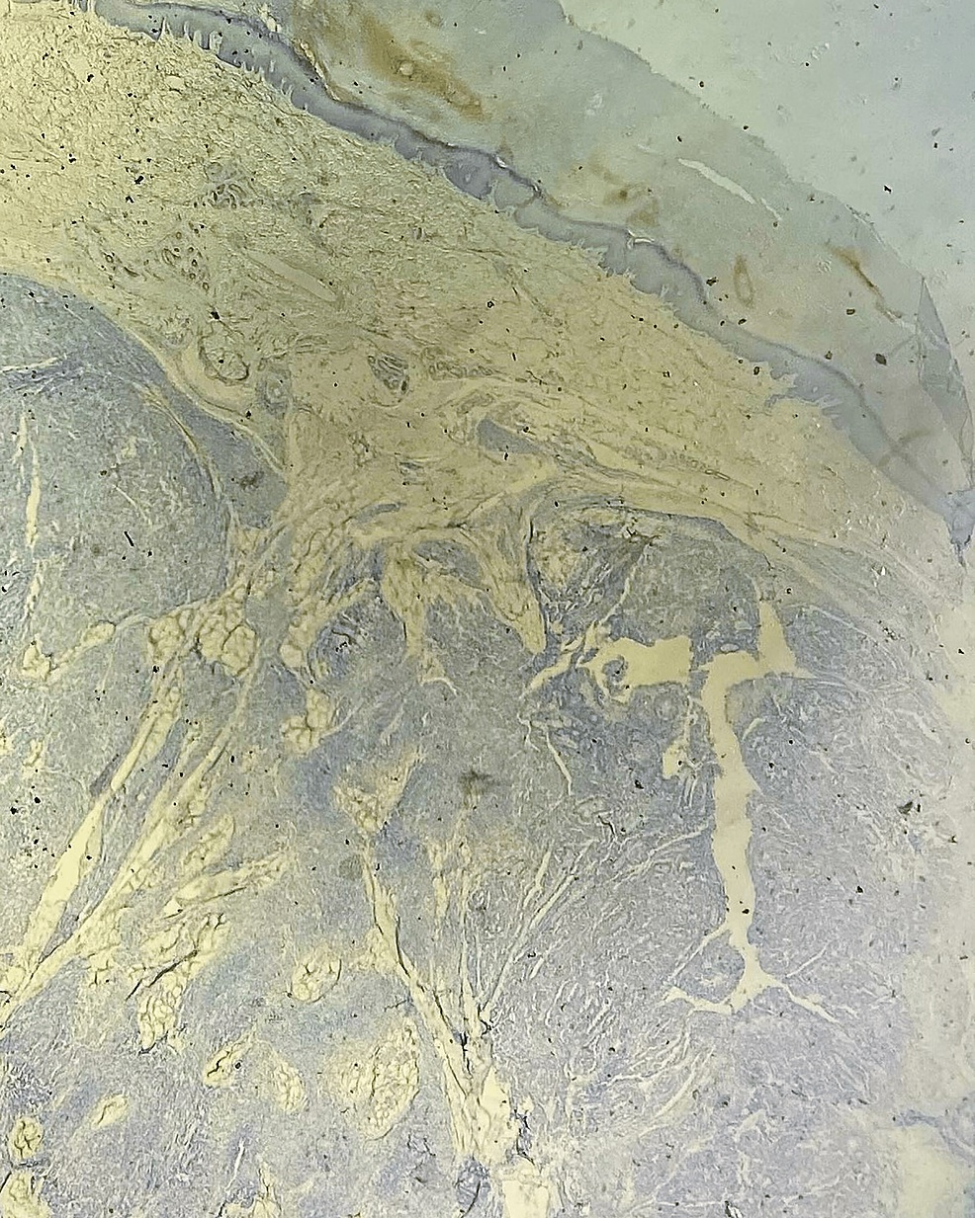
Immunostaining with P16 which is entirely negative (lost).

**Figure 6:**
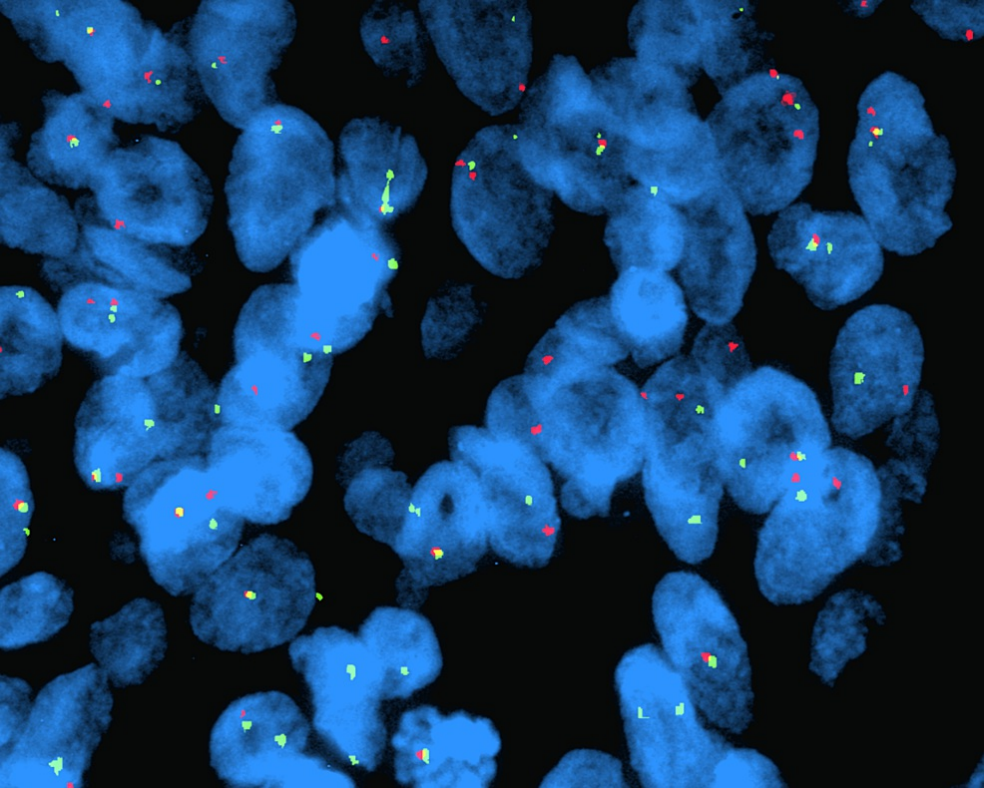
FISH analysis using Vysis LSI EWSR1 dual color break apart rearrangement probe for 22q12, showing EWS gene rearrangement.

A 31-year-old man presented with a 2.5 cm heel mass nodule. An MRI was performed, showing a grossly well-defined subcutaneous tumor of benign appearance compatible with an angioleiomyoma (Figure 1). Surgical excision of the mass was performed without a carcinological margin. The histopathologic examination showed a dermal and subcutaneous fat nodular mass of 23 mm with fascicles of spindle cells and cellular nests separated by fibrous bands. There was no epidermal component found with a grenz zone (Figure 2). Cells are fusiform or epithelioid with vesicular nuclei, prominent nucleoli, and clear-to-pale eosinophilic cytoplasm (Figure 3). Mitotic figures were found with some atypical ones. There was no necrosis area or lymphovascular invasion. Surgical margins were free by 1 mm. A first immunohistochemistry panel was performed on the tissue sections from formalin-fixed paraffin-embedded tissue (FFPE), including mesenchymal markers (CD34, CD31, SMA, Desmin, STAT6, and S-100 protein), all of which were negative except S-100 protein, and then we completed with melanic markers (HMB-45, Melan A), which were all diffusely positive, and P16 was lost (Figures 4-5). Accordingly, a diagnosis of melanoma was initially made. A meticulous clinical examination, as well as a complete radiological assessment, were carried out without having found a primitive site. However, this situation, including the young age of the patient and the absence of primary melanoma, led us to perform FISH on the specimen using the standard method for FFPE with a Dual Color Break Apart EWS Probe, allowing for the detection of EWS (22q12) gene rearrangement (Figure 6). Based on the FISH results, the young age, the location of the lesion, and the lack of an epidermal component, the diagnosis of cutaneous clear-cell sarcoma was approved. A re-excision was done for carcinological margins, and it was free of residual tumor cells.

## Discussion

Clear-cell sarcoma was first described in 1965 by F. Enzinger. It overlaps morphologically, immunohistochemically, and ultrastructurally with malignant melanoma, hence its name "soft tissue melanoma." Nevertheless, there are sufficient cytogenetic differences between cutaneous melanoma and clear-cell sarcoma to consider clear-cell sarcoma as a separate entity [1]. It is a rare mesenchymal malignant neoplasm, accounting for 1% of all soft tissue sarcomas. This tumor mainly affects young adults, between 20 and 40 years old (median age of 30 years). Women are the most affected [3]. This tumor commonly affects deep sites in the extremity, particularly the foot, ankle, hip, knee, thigh, and hand, with 50% of cases arising in the lower extremity, where they can be associated with tendons or aponeuroses [2]. Almost all patients present with a tumor mass of a few months to some year’s duration, less than 5 cm, circumscribe. Pain and tenderness are reported in approximately 30% to 50% of cases such as our patient [7]. The histopathological and immunohistochemical overlap between cutaneous clear cell sarcoma and nodular melanoma presents a challenging diagnosis to pathologists [8]. However, clear cell sarcoma affects the lower limbs of young patients, whereas melanoma arises in the sun-skin regions of older patients. Primary melanoma is typically more mitotically active and pleomorphic and is frequently associated with an epidermal component. CCS develops in deep tissues in association with aponeuroses or tendons, lacks cutaneous involvement, exhibits less cellular pleomorphism, and does not display an epidermal component. However, nodular, primary dermal, or metastatic melanoma presents a true diagnostic challenge since these tumors often lack an in situ component [9]. Histologically, at low magnification, clear-cell sarcoma shows well-demarcated nests or lobules and fascicles surrounded by fibrous bands continuing with oriented collagen structures. Cells are monomorphic, round, ovoid, or fusiform, and contain pale to clear or eosinophilic cytoplasm. The nuclei are typically round or ovoid, vesicular, dense at the periphery, and centered by a prominent, basophilic nucleolus. Tumor necrosis and mitoses are rare (usually less than 5 mitoses per 2 mm²). Multinucleated giant cells (where the nuclei are arranged in a crown) can be seen [1-3,10]. Immunohistochemically, CCS shares the same profile as melanoma; it diffusely expresses S-100 protein, SO, ×10, and also expresses HMB-45, Melan-A, Mel-CAM, and MiTF [1,11]. However, malignant melanoma (MM) is genetically different from CCS, which was demonstrated in the Yang et al. 2012 study [12], so they found that 51.6% of MM harbored BRAF mutations and 12.9% of MM harbored NRAS mutations. In contrast, none of the CCS samples showed BRAF or NRAS mutations [12]. These results were consistent with previous reports confirming that the mutation status of BRAF/NRAS may be a useful biomarker to distinguish MM from CCS. On the other hand, FISH evaluation using break-apart rearrangement probes specific for the EWSR1 gene on 22q13 shows EWSR1 gene rearrangement in up to 90% of cases. Withal, EWSR1 FISH has never been found rearranged in melanoma, and therefore, it is highly specific for CCS in this differential diagnosis [13]. This translocation rearranges the activating transcription factor-1 (ATF1) gene on 12q13 and the EWSR1 gene on 22q12, resulting in an EWSR1/ATF1 chimeric transcript. FISH represents a sensitive and cost- and time-efficient method for differentiating between CCS and MM, using a dual-color, break-apart probe in formalin-fixed, paraffin-embedded tissues.

Wide surgical excision with negative margins is the gold standard of CCS treatment. Adjuvant therapy is not necessary when the margins are clear. If the resection is close to the resection margins, adjuvant radiation is required. Chemotherapy is typically reserved for metastatic disease [3]. Prognostic factors are tumor size, site, necrosis, mitoses, and resection margins. So, long-term and close follow-ups are necessary because of late local recurrences and regional lymph nodes or distant metastases [14].

## Conclusions

The clinical presentation, histological, and immunohistochemical features of this case were compatible with melanoma. However, the young age and the absence of detected primary melanoma led us to perform fluorescence in situ hybridization that allowed for the detection of EWS (22q12) gene rearrangement which prompted a diagnosis of clear cell sarcoma. So, this case highlights the importance of considering the diagnosis of a clear-cell sarcoma in front of any dermal lesions with melanocytic features that do not have an epidermal component and of atypical presentation, especially in young patients, hence the interest in performing fluorescence in situ hybridization for EWSR1, which remains the key to the diagnosis of clear-cell sarcoma.
